# Short women with severe sepsis-related acute lung injury receive lung protective ventilation less frequently: an observational cohort study

**DOI:** 10.1186/cc10524

**Published:** 2011-11-01

**Authors:** SeungHye Han, Greg S Martin, James P Maloney, Carl Shanholtz, Kathleen C Barnes, Stacey Murray, Jonathan E Sevransky

**Affiliations:** 1 Critical Care Medicine Department, National Institute of Health, 10 Center Drive, Bethesda, MD 20892, USA; 2 Division of Pulmonary, Allergy and Critical Care, Emory University, 49 Jesse Hill Jr. Drive SE. Atlanta, GA 30303, USA; 3 Division of Pulmonary Sciences and Critical Care Medicine, University of Colorado, 4200 East 9th Avenue, Denver, CO 80262, USA; 4 Division of Pulmonary and Critical Care, University of Maryland, 22 S. Greene Street, Baltimore, MD 21201, USA; 5 Division of Allergy and Clinical Immunology, Johns Hopkins University, 5501 Hopkins Bayview Circle, Baltimore, MD 21224, USA; 6 Division of Pulmonary and Critical Care Medicine, Johns Hopkins University, 5501 Hopkins Bayview Circle, Baltimore, MD 21224, USA

## Abstract

**Introduction:**

Lung protective ventilation (LPV) has been shown to improve survival and the duration of mechanical ventilation in acute lung injury (ALI) patients. Mortality of ALI may vary by gender, which could result from treatment variability. Whether gender is associated with the use of LPV is not known.

**Methods:**

A total of 421 severe sepsis-related ALI subjects in the Consortium to Evaluate Lung Edema Genetics from seven teaching hospitals between 2002 and 2008 were included in our study. We evaluated patients' tidal volume, plateau pressure and arterial pH to determine whether patients received LPV during the first two days after developing ALI. The odds ratio of receiving LPV was estimated by a logistic regression model with robust and cluster options.

**Results:**

Women had similar characteristics as men with the exception of lower height and higher illness severity, as measured by Acute Physiology and Chronic Health Evaluation (APACHE) II score. 225 (53%) of the subjects received LPV during the first two days after ALI onset; women received LPV less frequently than men (46% versus 59%, *P *< 0.001). However, after adjustment for height and severity of illness (APACHE II), there was no difference in exposure to LPV between men and women (*P *= 0.262).

**Conclusions:**

Short people are less likely to receive LPV, which seems to explain the tendency of clinicians to adhere to LPV less strictly in women. Strategies to standardize application of LPV, independent of differences in height and severity of illness, are necessary.

## Introduction

Acute lung injury (ALI) is a serious clinical syndrome with a high case fatality rate characterized by acute hypoxemia with bilateral infiltrates on chest radiography in the absence of clinical evidence of left atrial hypertension [1-5]. Recent reports have suggested an increasing prevalence in the United States, up to 86.2 per 100, 000 person-years [3] and 2.2 cases per intensive care unit (ICU) bed per year [6]. The use of a lung protective ventilation (LPV) strategy has been shown to reduce mortality rates in intubated ALI patients [7].

Men and women have different mortality rates in ALI [8]. Several factors may explain this differential mortality rate. Women may have different incidence of ALI and thus different prevalence of ALI, or different case fatality rates from ALI than men. It has been reported that there is gender difference in genetic susceptibility to acute respiratory distress syndrome (ARDS) [9]. It is also possible that treatment or response to therapy may differ by gender. Differential care by gender in patients without ALI who received mechanical ventilation [10,11] has been reported.

The aim of this study was to investigate whether gender is associated with the use of LPV, and to identify the potential confounding factors associated with the use of LPV in an ongoing observational study of patients with sepsis-related ALI. We hypothesized that gender is not associated with the use of LPV in patients with sepsis-related ALI.

## Materials and methods

### Study population and design

As part of the Consortium to Evaluate Lung Edema Genetics (CELEG) study, invasively mechanically ventilated patients diagnosed with severe sepsis-induced ALI were prospectively enrolled from medical and surgical ICUs in seven academic medical centers between 2002 and 2008. Severe sepsis was defined according to Society of Critical Care Medicine/American College of Chest Physicians Consensus Definitions [12]; ALI was defined as mechanically ventilated patients who met the American-European Consensus Definitions [13]. Exclusion criteria were allogeneic bone marrow transplant and severe leukopenia (white blood count < 1, 000/μL). All ICU patients were screened daily by specially trained research staff with previous experience in ALI trials, and approached if they were eligible. The details of the CELEG study have been described elsewhere [14]. The study was approved by the institutional review boards of all participating centers, and informed consent was obtained from the patients or surrogates. All severe sepsis-related ALI subjects with available heights were selected for our analyses.

### Outcomes and exposures

The primary outcome was the use of LPV during the first two days after developing ALI. We used an algorithm based on tidal volume (mL/kg of predicted body weight (PBW)), arterial pH and plateau pressure (cmH_2_O) on the day of ALI onset, derived from the ARDSNet ventilation protocol [7] (See Table 1). In constructing this algorithm, we chose the lowest arterial pH on the day of ALI onset to consider cases where the tidal volume would have been increased up to 8 mL/kg of PBW to treat the patient's severe acidosis (49 patients). Patients who received LPV within two days of ALI onset were considered to have received LPV. All decisions regarding ventilatory strategy were made by primary treating teams.

**Table 1:** Definition of lung protective ventilation (LPV)

V_T_0 ^a^ (mL/kg PBW)	Plateau pressure ≤ 30 cm H_2_O				Plateau pressure > 30 cm H_2_O				Plateau pressure, missing			
	V_T_1 ^b ^(mL/kg PBW)				V_T_1 ^b ^(mL/kg PBW)				V_T_1 ^b ^(mL/kg PBW)			
	≤ 6.5	(6.5, 8]^c^	> 8	missing	≤ 6.5	(6.5, 8]^c^	> 8	missing	≤ 6.5	(6.5, 8]^c^	> 8	missing
**≤ 6.5**	LPV	LPV	LPV if pH < 7.15	LPV	LPV if pH < 7.15 or (TV0 > TV1)	LPV if pH < 7.15	No	LPV if pH < 7.15	LPV	LPV	None	LPV
			No if pH ≥ 7.15		No if pH ≥ 7.15 and (TV0 ≤ TV1)	No if pH ≥ 7.15		No if pH ≥ 7.15				
**(6.5, 8] ^c^**	LPV	LPV	LPV if pH < 7.15	LPV	LPV	LPV if pH < 7.15	No	LPV if pH < 7.15	LPV	LPV if pH < 7.15	No	LPV if pH < 7.15
			No if pH ≥ 7.15			No if pH ≥ 7.15		No if pH ≥ 7.15		No if pH ≥ 7.15		No if pH ≥ 7.15
**> 8**	LPV	LPV if pH < 7.15	No	No	LPV	LPV if pH < 7.15	No	No	LPV	LPV if pH < 7.15	No	No
		No if pH ≥ 7.15				No if pH ≥ 7.15				No if pH ≥ 7.15		

^a ^V_T_0; tidal volume on the day of ALI onset, ^b ^V_T_1; tidal volume on one day after ALI onset, ^c ^(6.5, 8]; higher than 6.5 AND less than or equal to 8

The exposures we considered are listed in Table 1: patient-related factors (age, gender, self-reported ethnicity, height, and body mass index) and severity of illness (Acute Physiology and Chronic Health Evaluation (APACHE) II score [15] and the ratio of partial pressure of arterial oxygen (PaO_2_) and fraction of inspired oxygen (FiO_2_)). The first PaO_2_/FiO_2 _ratio at the time of ALI diagnosis was used for our analyses.

### Statistical analysis

Descriptive statistics were reported with mean ± standard deviation (SD) for continuous variables and proportions for categorical variables, and were analyzed by Mann-Whitney and chi-square tests, respectively. We selected biologically plausible variables with *P*-values < 0.05 in univariable analyses, and estimated odds ratio (OR) of receiving LPV in multivariate models. To account for the possibility that the prescription of tidal volumes in the same hospital may not be independent, we ran a logistic regression model with robust and cluster options. Sensitivity analysis was performed to evaluate the potential variability of the results caused by missing data. All analyses were performed using Stata Statistical Software: Release 10 (StataCorp. 2007. College Station, TX, USA: StataCorp LP).

## Results

**Figure 1:**
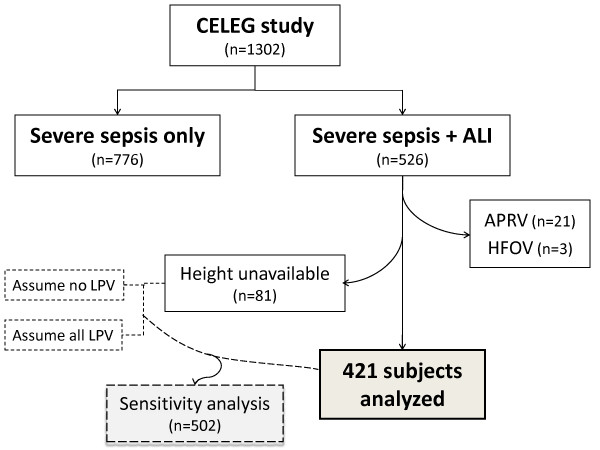
**Outline of the study**. ALI, acute lung injury; APRV, airway pressure release ventilation; CELEG, Consortium to Evaluate Lung Edema Genes study; HFOV, high frequency oscillation ventilation.

Among 526 patients with sepsis-related ALI in the CELEG study, 24 patients were excluded because they were not on a fully controlled mode of mechanical ventilation with measured tidal volume: 21 subjects on airway pressure release ventilation and 3 subjects on high frequency oscillation ventilation. An additional 81 patients could not be evaluated as their heights were unavailable. Therefore, a final sample of 421 sepsis-related ALI subjects was used for our analyses (Figure 1).

Our study sample had a mean age of 54.9 ± 17.3 years, with 57% men and 68% European-Americans. The mean APACHE II score was 27.7 ± 7.9 and the mean PaO_2_/FiO_2 _ratio was 131 ± 63. Actual body weight (ABW) was 19 kg heavier than PBW (84 kg versus 66 kg, *P *< 0.001), and the majority (79%) of the patients had higher ABW than PBW. Women in our study were shorter and had higher APACHE II scores compared with men (Table 2).

**Table 2:** Baseline characteristics by gender

Characteristic	Men (n = 238)	Women (n = 183)	*P*
**Age (year)**	55 ± 17	55 ± 17	0.71
**Ethnicity (%)**			
**European-American**	64	62	
**African-American**	36	38	0.80 ^†^
**Height (cm)**	177 ± 8	162 ± 7	< 0.001
**Body mass index (kg/m^2^)^a^**	28 ± 8	29 ± 9	0.65
**APACHE II score**	27 ± 8	29 ± 8	0.01
**PaO_2_:FiO_2 _^b^**	135 ± 63	126 ± 63	0.08

APACHE II, Acute Physiology and Chronic Health Evaluation II

*Continuous variables are reported as mean ± SD, and compared by Mann-Whitney test

† By Chi-square test

^a ^n = 419, ^b ^n = 416

A total of 225 (53%) subjects received LPV. Women received LPV less frequently than men (46% versus 59%, *P *< 0.001). In contrast, 307 (73%) subjects were categorized as receiving LPV based on ABW, without gender difference (75% and 72% for women and men, respectively, *P *= 0.416). Height, gender and severity of illness as measured by APACHE II scores were significantly associated with the use of LPV in univariable analyses. In multivariable analysis, height and APACHE II score remained significantly associated with the use of LPV. However, gender was not associated with the use of LPV in multivariable analysis (*P *= 0.262) (Table 3).

**Table 3:** Factors associated with LPV

	Univariate Odds ratio (95% CI)	Multivariate Odds ratio (95% CI)
**Women vs. Men**	0.58 (0.43 to 0.79) *	1.40 (0.78 to 2.54)
**APACHE II**	1.04 (1.01 to 1.07) *	1.06 (1.03 to 1.09) *
**Height**	1.05 (1.03 to 1.08) *	1.07 (1.03 to 1.11) *

APACHE II, Acute Physiology and Chronic Health Evaluation II; CI, confidence interval * *P *< 0.001

**Figure 2:**
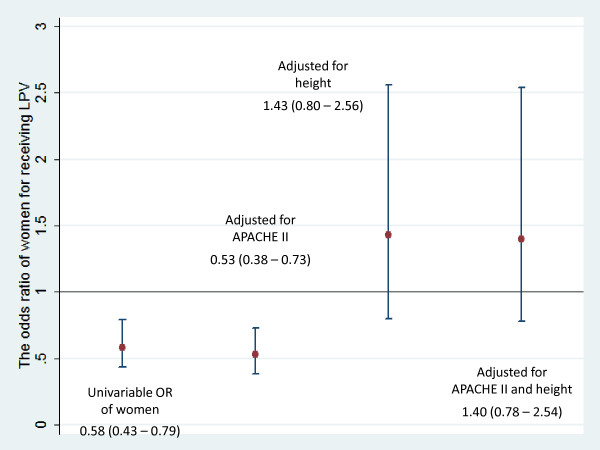
**Association of gender (women versus men) with the use of LPV: OR (95% CI)**. APACHE II, Acute Physiology and Chronic Health Evaluation II; CI, confidence interval; LPV, lung protective ventilation; OR, odds ratio.

In order to investigate further the association between gender and the use of LPV with its confounding factors, additional logistic regressions were fitted. The ORs of receiving LPV for women versus men in different models were showed in Figure 2. In the univariate model, the relative odds of receiving LPV comparing women to men was less than 1, which means women were less likely to receive LPV than men. Including the APACHE II score in the model did not change the direction and significance of the association between gender and the use of LPV. However, after adjustment for height and/or APACHE II score, the gender was no longer associated with the use of LPV (*P *= 0.231 and 0.262, respectively).

In order to see how much our findings could be potentially biased by missing data, we performed a sensitivity analysis with two extreme scenarios - 1) all the subjects with missing height received LPV and 2) all the subjects with missing heights did not receive LPV - and saw how much results were changed in the two scenarios (Table 4). Missing heights were replaced with average heights of men and women, respectively, in our study population. The results were similar with the exception of the OR for women versus men in a multivariate model under the assumption of no LPV. Height remained significantly associated with the use LPV throughout all the models.

**Table 4:** Sensitivity analysis including the subjects with missing values (n = 502): OR (95% CI)

	Assumption of all LPV in subjects with missing height		Assumption of no LPV in subjects with missing height	
	
	Univariate	Multivariate	Univariate	Multivariate
Women vs. Men	0.58 (0.48 to 0.71)***	1.45 (0.82 to 2.54)	0.68 (0.49 to 0.95)*	1.74 (1.30 to 2.33)***
APACHE II	1.03 (1.01 to 1.05)**	1.04 (1.02 to 1.07)***	1.04 (1.02 to 1.07)**	1.05 (1.03 to 1.08)***
Height	1.05 (1.03 to 1.08)***	1.07 (1.03 to 1.12)**	1.05 (1.02 to 1.07)***	1.07 (1.04 to 1.11)***

APACHE II, Acute Physiology and Chronic Health Evaluation II; CI, confidence interval; OR, odds ratio

* *P *< 0.05, ** *P *< 0.01, *** *P *< 0.001

## Discussion

Our prospective observational study at seven academic centers revealed that women were approximately 40% less likely to receive LPV compared to men during the first two days after ALI onset. This differential exposure may be explained by their height difference. According to our multivariable analysis, severe sepsis-related ALI patients were 20% more likely to receive LPV if they were one inch (2.54 cm) taller, while gender was not associated with the use of LPV. Our finding suggests that intensive care regarding mechanical ventilation in ALI patients may be influenced by patient-related factors such as height and severity of illness.

Several critical illnesses have different outcomes by gender. Women have higher mortality than men after developing acute myocardial infarction [16], respiratory distress requiring mechanical ventilation [17] and nosocomial infections [18], while male patients with blunt trauma experience a higher risk of death than females [19]. Several potential explanations for these mortality differences include physiologic and hormonal differences [20-23], or differential exposure to intensive care and treatments. Men have been shown to be more likely to be admitted to ICU and undergo aggressive measures, such as mechanical ventilation, vasoactive medication, renal replacement therapy, or central vascular catheterization compared with women, after adjustment for severity of illness [24,25]. It has also been reported that the adjusted rates of reperfusion therapy and coronary angiography after myocardial infarction were higher in men than in women [26].

Our study showed the ventilatory care in intubated ALI patients was different by gender, likely from their height difference. On average, women are shorter than men and thus their calculated tidal volumes in mL would be smaller than men's, based on PBW derived from height and gender. If this factor is not considered in the ventilatory care of women with ALI and consequently women receive higher tidal volumes than men, this difference in treatment may contribute to the higher mortality seen in mechanically ventilated women compared with men [17]. Another possibility is that women may be under-diagnosed with ALI and get LPV less often than men. Unfortunately this cannot be confirmed from our data. There was, however, no gender difference in the use of LPV if it was defined by ABW rather than PBW. This suggests the decreased use of LPV in women may be more related to the differential treatment itself rather than differential diagnosis.

We found that only 53% of severe sepsis-related ALI patients received LPV. This similar lack of compliance with the use of LPV has been reported in other studies [27-29]. Several factors have been reported as barriers for initiating and maintaining LPV, including concerns about the patient's hypercapnia/acidosis [28,30] and difficulty in calculating correct tidal volumes based on PBW [30]. Even though our algorithm considered the cases where tidal volumes increased to treat severe acidosis, approximately half of ALI patients did not receive LPV during the first two days after ALI onset. Since height was not always available in our study population, it is likely that some patients received tidal volumes based on other factors, such as actual body weight. Use of actual rather than predicted body weight may be associated with unintended larger tidal volumes since PBW has been reported to be smaller than ABW [10]. The higher use of LPV based on ABW seen in our study also supports this possibility.

Our multivariate analysis showed that a one-point increase in APACHE II score was associated with a 6% higher using rate of LPV in severe sepsis-induced ALI patients. Less ill patients may be under-diagnosed or delayed-diagnosed and, thus, received LPV less frequently for the first two days after ALI onset. In addition, clinicians may be more likely to prescribe LPV in patients with higher initial plateau pressures. Further, since oxygenation is a part of APACHE II score calculation, it is also possible that the degree of lung injury itself influenced the use of LPV. In our study population, lower PaO_2_/FiO_2 _ratio and higher APACHE II score were all significantly associated with higher using rate of LPV in both univariate and multivariate models. Patients with a PaO_2_/FiO_2 _ratio < 200 were twice more likely to receive LPV than those with a ratio of 200 or more (data not shown).

Our study additionally suggests that a written protocol alone, which has been reported to be associated with increased use of LPV in ALI patients [27], may not be enough to increase the compliance of LPV fully. Although most of our study centers, except the University of Maryland (n = 41), implemented written protocols for LPV during the study period, the use of LPV was not obviously satisfying (57% in the six centers with protocols). Extra-tools to enhance the use of LPV, such as incorporating a reminder to record height and PBW may be necessary [31].

Our study has several limitations. We only have the ventilatory data during the first two days after ALI onset, rather than the repeated measures throughout the course of the disease. It is possible that the first two days of data may not be enough to determine LPV as the ventilatory setting could be changed later. However, ventilatory settings within 48 hours after ALI onset are known to be important predictors for outcomes [32,33]. Another limitation is that 16% (81 out of 502) of the ALI patients who received the conventional assist-control mode of mechanical ventilation in our CELEG study had missing heights, so that they were not included in our analyses. Patient characteristics, such as age, gender and weight, and severity of illness, such as APACHE II scores and PaO_2_/FiO_2 _ratio, were similar between ALI patients with available heights and those with missing heights. However, outcomes such as 28- and 60-day mortality and ventilator free days were worse in the patients with missing height compared to those with available height, possibly because they were ventilated with higher tidal volumes calculated with actual weights or other factors rather than PBW. This suggests missing data of LPV might not be at random and, thus, potentially bias our findings. However, our sensitivity analysis with two extreme assumptions of LPV did not show any substantial changes in the results compared to our original analyses, and gives us the same inferences.

## Conclusions

In conclusion, women are less likely to receive LPV compared to men. However, after adjustment for height and severity of illness, there is no difference between men and women in exposure to LPV. This is most likely from the differences in height, leading to the inaccurate selection of tidal volumes for women. Strategies to standardize LPV delivery, independent of differences in severity of illness and height, are necessary.

## Key messages

• Short patients were less likely to receive lung protective ventilation during the first two days after severe sepsis-related acute lung injury onset. This led to the inaccurate selection of tidal volumes for women and, thus, resulted in 40% lower rate of receiving lung protective ventilation in women compared to men.

• Overall compliance with the use of lung protective ventilation in acute lung injury patients was low even in the presence of written protocols.

• Strategies to standardize lung protective ventilation delivery, independent of differences in patient factors, such as height and severity of illness, are necessary.

## Abbreviations

ABW: actual body weight; ALI: acute lung injury; APACHE II: Acute Physiology and Chronic Health Evaluation II; CELEG: Consortium to Evaluate Lung Edema Genetics; CI: confidence interval; FiO_2_: fraction of inspired oxygen; ICU: intensive care unit; LPV: lung protective ventilation; OR: odds ratio; PaO_2_: partial pressure of arterial oxygen; PBW: predicted body weight; SD: standard deviation; V_T_0: tidal volume on the day of ALI onset; V_T_1: tidal volume on one day after ALI onset.

## Competing interests

The authors declare that they have no competing interests.

## Authors' contributions

SH conceived the study, ran the statistical analysis and drafted the manuscript. GSM assisted with the design of the study and made substantial revisions to the final manuscript. JPM, CS, KCB and SM made substantial revisions to the final manuscript. JES assisted with the design of the study, drafted the manuscript, and made substantial revisions to the final manuscript. All authors have read and approved the manuscript for publication.
